# The Effect of ECAP Processing Conditions on Microstructural Evolution and Mechanical Properties of Pure Magnesium—Experimental, Mathematical Empirical and Response Surface Approach

**DOI:** 10.3390/ma15155312

**Published:** 2022-08-02

**Authors:** Abdulrahman I. Alateyah, Waleed H. El-Garaihy, Majed O. Alawad, Samar El Sanabary, Sally Elkatatny, Hany A. Dahish, Hanan Kouta

**Affiliations:** 1 Department of Mechanical Engineering, College of Engineering, Qassim University, Unaizah 56452, Saudi Arabia; a.alateyah@qu.edu.sa; 2 Mechanical Engineering Department, Faculty of Engineering, Suez Canal University, Ismailia 41522, Egypt; sally_mahmoud@eng.suez.edu.eg; 3 Materials Science Research Institute, King Abdulaziz City for Science and Technology (KACST), Riyadh 12354, Saudi Arabia; 4 Department of Production Engineering and Mechanical Design, Port Said University, Port Fuad 42526, Egypt; samar.abaas@eng.psu.edu.eg (S.E.S.); hanan.kamel@eng.psu.edu.eg (H.K.); 5 Department of Civil Engineering, College of Engineering, Qassim University, Unaizah 56452, Saudi Arabia; ha.dahish@qu.edu.sa; 6 Civil Engineering Department, Faculty of Engineering, Fayoum University, Fayoum 63514, Egypt

**Keywords:** severe plastic deformation, equal channel angular pressing, pure magnesium, microstructural evolution, response surface methodology, optimization

## Abstract

In this study, a quantitative evaluation approach was used to investigate how certain ECAP processing parameters affect the microstructural evolution, Vicker’s microhardness values and tensile properties of pure Mg. The ECAP processing parameters were number of passes, ECAP die channel angle and processing route type. The response surface methodology (RSM) technique was used to design 16 runs of the experiment using Stat-Ease design expert software. Billets of pure Mg were processed up to four passes of routes Bc, A and C at 225 °C. Two ECAP dies were used with internal channel angles of 90° and 120°. Experimental findings were used to establish empirical models to assess the influence of the ECAP processing parameters on grain size and mechanical properties of ECAPed billets. The established relationships were examined and validated for their adequacy and significance using ANOVA as well as several statistical criteria. Response surface plots and contour graphs were established to offer better understanding of the intended relationships. In addition, the optimum processing parameters for grain size, hardness values and tensile properties were defined. Both experimental results and the theoretical model revealed that route Bc is the most effective route in grain refining. The experimental findings showed that four passes of route Bc through the die channel angle 90° revealed a significant reduction in the grain size by 86% compared to the as-annealed counterparts. Similar to the grain size refining, four-passes processing through the ECAP die with an internal channel angle of 90° leads to improved Vicker’s microhardness values. Additionally, four passes of route Bc using the 90° die angle recorded a significant HV increase at the edge and central areas by 112% and 78%, respectively, compared to the as-annealed counterpart. On the other hand, according to the optimization findings, two passes of route Bc using a die angle of 120° resulted in the best ultimate tensile strength for pure Mg, whereas four passes of route Bc revealed the optimum ductility at fracture.

## 1. Introduction

Magnesium (Mg) and its alloys are widely considered ultra-light alloys due to their superior strength to weight ratio. Their densities are two thirds, and one quarter, respectively, of that of aluminum alloys and steel [1,2,3]. Mg alloys display unique properties, such as high specific stiffness, good recyclability and excellent specific strength, which increase the demand for them in transportation industries [4,5,6,7,8,9]. Furthermore, the usage of Mg alloys in automotive is an effective means of reducing carbon dioxide emissions and fuel usage. Mg alloys’ high specific strength results in decreased car weights; reducing a car’s weight by 10%, for example, results in saving 5–10% fuel [10]. However, the most significant disadvantage of employing Mg alloys is their low formability. This consequently diminishes their performance in automobile applications.

The available deformation modes of Mg alloys are restricted due to the nature of the Mg crystal’s hexagonal close-packed (HCP) structure [11]. Namely, the significant discrepancy in resolved shear stresses between the slip systems decreases the deformability of Mg alloys [5,12,13,14]. Therefore, Mg alloys fracture when subjected to traditional deformation methods, such as extrusion and rolling at room temperature [15]. However, Mg alloys’ deformability increases at higher temperatures. The drawback to hot working is that the dynamic recrystallization and recovery processes associated with it mitigate the effects of deformation hardening [16,17,18]. Several fruitful efforts have been made to develop Mg alloys with useful strength–ductility combinations [19,20] and with better corrosion resistance [20,21]. These attempts to improve Mg alloys’ formability showed that controlling the texture using plastic deformation techniques is a well-suited approach to formability enhancement. 

Severe plastic deformation (SPD) techniques have great potential for ambient temperature deformation of Mg alloys [22,23]. SPD includes several techniques applicable to a wide range of materials; one such technique is equal channel angular pressing (ECAP) [24,25,26,27]. ECAP possesses several desirable features: its ability to produce ultra-fine-grain (UFG) structures, its capability to fabricate nanostructures efficiently and its applicability to industry [28,29,30,31].

Several studies have been investigated to study the ECAP process parameters and their impact on deformation behavior as the mechanical and microstructural characteristics depend on the degree of plastic deformation. The equivalent strain ($\varepsilon_{eq}$) can be theoretically modeled as a function of the die geometry using Equation (1) [31,32]. The strain is affected by the internal channel angle (ϕ), the outer corner angle (Ψ) and number of passes (N).

(1) $$\varepsilon_{eq}=\frac{N}{\sqrt{3}}\;\left[2\;cot\left(\frac{\varphi +\Psi }{2}\right)+\Psi \;cosec\left(\frac{\varphi +\Psi }{2}\right)\right]$$

**Figure 1:**
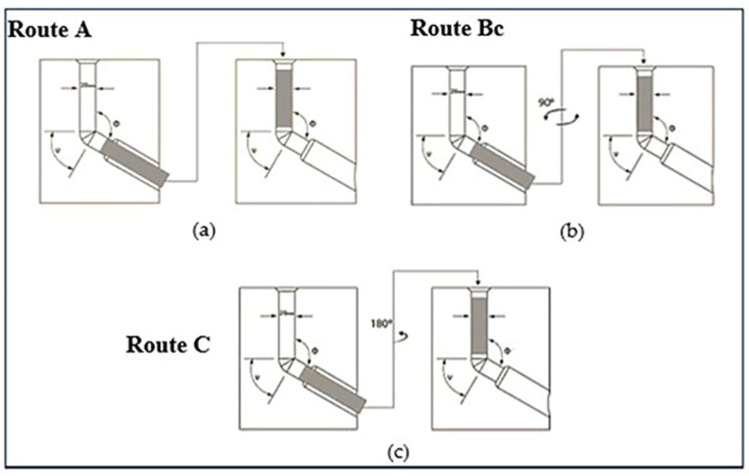
Schematic of the different routes of ECAP through multiple passes; (**a**) route A, (**b**) route Bc, and (**c**) route C.

Furthermore, the ECAP strain is affected by whether the processed material billets were rotated along its longitudinal axis between passes or not. Different combinations of billet rotations define the ECAP routes. The common ECAP routes are route A, Bc and C. Figure 1 presents the differences among the different ECAP routes. In route A, the rod is not rotated. The rod in route Bc is rotated by 90° between passes. Finally, in route C, the billet is rotated by 180° between subsequent passes [33]. ECAP processing using multiple passes and different routes produces the most grain refinement but also results in significant changes in the shear plane over the entire process [34]. 

Previous research shows that the compressive mechanical properties of pure Mg deteriorated after the second ECAP pass at room temperature using route Bc and ϕ = 90°. This reduction is explained by the activation of the non-basal slipping systems, and by the newly formed texture. However, the mechanical properties improved after the fourth pass due to grain refinement [35]. In addition, Venkatachalam et al. [36] applied the ECAP process to the AA2014 aluminum alloy. The mechanical properties were substantially enhanced by processing using route Bc compared to processing via other routes A, C and Ba as the effective strain was homogenized among all the planes. A.I. Alateyah et al. [37] studied the effect of different internal channel angle on the strain homogeneity, microstructural evolution, crystallographic texture and mechanical properties of pure magnesium. The study was conducted experimentally using ϕ = 90° and 120° on samples processed for four passes through route Bc at 225°. A numerical finite element analysis approach was conducted using Simufact-forming software. FE simulation showed that the ϕ = 90 sample had a more homogenous distribution of stress compared to the 120° one. In addition, investigation of the microstructural evolution and mechanical properties revealed that the ϕ = 90° sample showed stronger texture and a higher ultimate strength than the 120° one. 

Previous research inquiries optimized ECAP analysis using response surface methodology (RSM). RSM is an empirical set of mathematical and statistical tools that can be used to build, modify and optimize the processes. RSM combines real and modelled responses’ efficiency behavior with a set of effective parameters based on their individual and interactive effects. The RSM is optimized using a genetic algorithm (GA) that avoids localized optimum point uncertainty [38].

Daryadel [39] simulated the ECAP process on the 7075 aluminum alloy with copper casing using the finite element method. RSM was used to design 31 experiments to investigate the effect of four processing (explanatory) parameters on the maximum required force and strain. The simulated results were verified by comparing the experimental and simulation maximum force. On the maximum required force and strain, the effects of four ECAP parameters (channel angle, corner angle, friction coefficient and casing thickness) were investigated. The regression models for computing the maximal forming force and strain are illustrated using analysis of variance (ANOVA). The author concluded that channel angle affects resultant force the most. Similarly, the strain was affected by channel and corner angle, and the friction coefficient and the thickness of copper on strain had no significant effect. Finally, the predicted optimal ECAP conditions for reducing the maximum forming force and increasing the strain were 93.64°, 0°, 0.001 and 1.62 mm for channel angle, corner angle, friction coefficient and casing thickness, respectively. Abbas et.al. [40] investigated the machining processes of recycled billets Al6061 chip. The billets underwent a three-step process: cold compact, hot extrusion and, finally, ECAP to improve the mechanical properties. Surface roughness and metal removal rate were measured as performance characteristics against various cutting parameters and the number of ECAP passes. The authors concluded that feed was the most influential parameter on the generated surface. Optimum values of MRR and generated surface roughness were obtained using a desirability function approach. The minimum values of surface roughness and maximum MRR were obtained at a cutting velocity of 195 m/min, a feed of 0.073 mm/rev, a depth of cut of 0.4 mm and three passes of ECAP process.

Under RSM, central composite design (CCD) is used for modelling and optimization. To optimize the response performance of any process without localized uncertain optimum point confusion, CCD, desirability function (DF) and GA can be used [38]. Many studies have used GA and hybrid RSM-GA to optimize the process condition. Deshwal et al. [41] investigated the tensile strength of a PLA-fabricated part produced by fused deposition modeling (FDM)—a powerful 3D printing technique—using a statistical technique equipped with numerous hybrid static tools. The contribution of each of the FDM parameters (infill density, temperature and speed) on the maximum performance tensile strength was investigated. To optimize FDM process parameters, hybrid optimization approaches, such as genetic algorithm-artificial neural network (GA-ANN), genetic algorithm-response surface methodology (GA-RSM) and genetic algorithm-adaptive neuro fuzzy interface system (GA-ANFIS), are used. The author concluded that the percentage accuracies of GA-ANN, GA-RSM and GA-ANFIS are 99.89%, 99.3% and 99.55%, respectively. Moreover, the maximum tensile strength of PLA obtained by GA-ANN was 47.0212 MPa, and the optimal conditions were infill density 100 %, temperature 210°C and speed 124.778 mm/s.

A limited number of studies examining ECAP performance numerically were found. However, the experimental studies investigating the effect of ECAP process on pure Mg are limited due to its poor deformability, and mostly focus on ECAP of Mg alloys at ϕ of ≤90°. Therefore, the present study investigates the ECAP conditions necessary for optimum performance characteristics. These characteristics are grain size (G_R_), hardness measurement at two positions of specimen: center (H_C_) and edge (H_E_), and tensile characteristics, namely ultimate tensile strength (σ_u_) and ductility (D). In addition, an experimental investigation is conducted on pure Mg under various ECAP conditions, such as number of passes (N), ECAP die angle (ϕ) and the type of processing route. A complete analysis of ECAP’s effect on microstructural evolution and mechanical properties was considered. The research’s design is based on RSM, which is used to identify the optimum ECAP parameter levels by analyzing the impact of ECAP conditions on responses. A second-order regression model and analysis of variance (ANOVA) were created to analyze the ECAP condition of optimum responses. GA was applied to optimize the ECAP condition. Finally, hybrid RSM-GA was created to improve the optimization of ECAP responses, and corresponding conditions were evaluated using GA.

## 2. Materials and Methods

### 2.1. Experimental Design

In this study, the effect of 3 ECAP parameters on pure Mg’s microstructure and mechanical properties are investigated. The 3 parameters are: number of passes (N), ECAP die angle (ϕ) and the type of processing route, as shown in Table 1. In this investigation, the combinations of ECAP parameter values used were designed using response surface methodology (RSM). A total of 16 runs were carried out to investigate various ECAP responses. The explanatory variables are grain size (G_R_), hardness measurements at the center (H_C_) and edge (H_E_) of the specimen and tensile characteristics—namely ultimate tensile strength (σ_u_) and ductility (D). Experimental data obtained from the microstructural evolution, Vicker’s hardness and tensile tests are shown in Table A1 in Appendix A. The RSM technique was used to analyze three factors with a small number of tests in order to model a second-order response surface. After that, the genetic algorithm is employed to figure out which fitness value is the best.

### 2.2. Material and Methodology

In the present study, rolled billets of pure Mg 20 mm in diameter and 60 mm in length were used. Before ECAP processing, the billets were annealed at 250 °C for 1h, followed by furnace cooling. The pure Mg billets were deformed by ECAP at 225 °C with different numbers of passes (1, 2 and 4 passes) through different routes (A, Bc and C), with 0.05 mm/s ram speed. The ECAP die consists of two channels intersecting with an outer die angle (Ψ) of 20° and with different inner angles ϕ (90° and 120°). A graphite-based lubricant was used to reduce friction between the billets and die walls. The samples after ECAP processing were centrically cut along their LS along the plane perpendicular to the die entry channel and parallel to the flow plane (pressing direction). The reference axes were labeled according to the ECAP direction: the transversal direction “X” (TD), the normal direction “Z” (ND) and “Y” (ED).

Microstructural and crystallographic texture of Mg billets after ECAP were studied using a field emission scanning electron microscope (FESEM, Hitachi, Ltd., Tokyo, Japan) that is equipped with a NordlysMax2 electron back-scatter diffraction (EBSD) detector. EBSD was performed on the top surface, the TD-ED plane, using a SU-70 SEM (Hitachi, Ltd., Tokyo, Japan) operating at 15 kV and with a typical current of 1.5 nA. The samples’ surfaces were prepared before the EBSD by grinding and mechanically polishing down to 1 µm using a tripod polisher, then polished chemically with colloidal silica (0.05 µm) for 24 h. by a BUEHLER Vibrometer (Buehler, Tucson, AZ, USA). 

Mechanical characterization of the ECAPed samples was measured using the Vickers micro-hardness tester (Shimadzu HMV-FA) under a load of 1 kg for a loading time of 15 s. Additionally, tensile properties at room temperature were measured using uniaxial tensile tests conducted by a universal testing machine (Instron 4210, Norwood, MA, USA) at a strain rate of 10^−3^ s^−1^. Dimensions of tensile samples were set based on the E8M/ASTM standard. Three specimens per ECAP processing condition were tested to confirm the accuracy of the results. Tensile specimens were sectioned from the center of the ECAPed rods.

### 2.3. Regression Model

Response surface methodology (RSM) is a powerful tool used to formulate, model, analyze, design and improve optimization processes by a set of statistical and mathematical tools. RSM consists of three main steps. First, conducting multiple runs of the experiment technique with different ECAP process parameters values. Second, developing an appropriate interaction between ECAP response and input factors by regression modelling. Finally, optimizing to identify the contribution of each ECAP process parameter on the appropriate output ECAP response [42,43]. The regression model provides the interaction between the process response and explanatory variables by fitting them into a second-order polynomial equation [44]. 

Stat-Ease Design Expert software (version 13.0.5, Stat-Ease, Inc., Minneapolis, MN, USA) is a useful tool for optimizing complicated systems for scientific and industrial applications and was, therefore, used to analyze the experimental data [45,46]. The regression transformations available from Design Expert include linear, square root, natural logarithm, logarithmic with base 10, inverse square root, inverse, power, logit and arcsine square root.

In this study, ECAP parameters were classified into numeric parameters, such as N and φ, and categorical parameters, such as processing route. To transform the categorical parameters to numerical ones, dummy coding and binary coding were utilized. In many forms of estimating models, such as linear regression, dummy coding is one approach of using categorical predictor variables. It also uses only ones and zeros to transmit all of the necessary information regarding group membership. Dummy variables are the coded categories that are generated for a category variable. A combination of K-1 dummy variables is required to develop mutually exclusive and comprehensive dummy variables that represent a certain categorical variable with K groupings [47]. For categorical variables route A, route Bc and route C, the dummy variables were coded by X_1_ and X_2_, as shown by matrix in Equation (2). If X_1_ = 1 and X_2_ = 0, then the route type is A; if X_1_ = 0 and X_2_ = 1, then the route type is Bc and if X_1_ = 0 and X_2_ = 1, then the route type is C.

(2) $$\begin{array}{cc} {\;X}_{1} & X_{2} \end{array}\;\begin{array}{c} A\\ Bc\\ C \end{array}=\left[\begin{array}{cc} 1 & 0\\ 0 & 1\\ 0 & 0 \end{array}\right]$$

Many trails of regression transformation from and interaction between independent variables were examined in order to model the output responses of ECAP. The best regression coefficient (R^2^) indicates that the models generated in the experimental research are statistically significant.

### 2.4. Genetic Algorithm (GA)

Genetic algorithms (GA) have been used in science and engineering as an intelligent and efficient method for addressing real-world optimization problems in a cost-effective approach. In each individual, the genetic algorithm generates an optimization algorithm and a random number of generations, whereas conventional algorithms generate only a single point and use a deterministic rule to determine the next generation. Every individual’s fitness function is assessed in each generation. The GA uses criteria to determine a global minimum value for a fitness function and ensures that the result converges [43,48].

## 3. Results and Discussion

### 3.1. Microstructural Evolution

**Figure 2:**
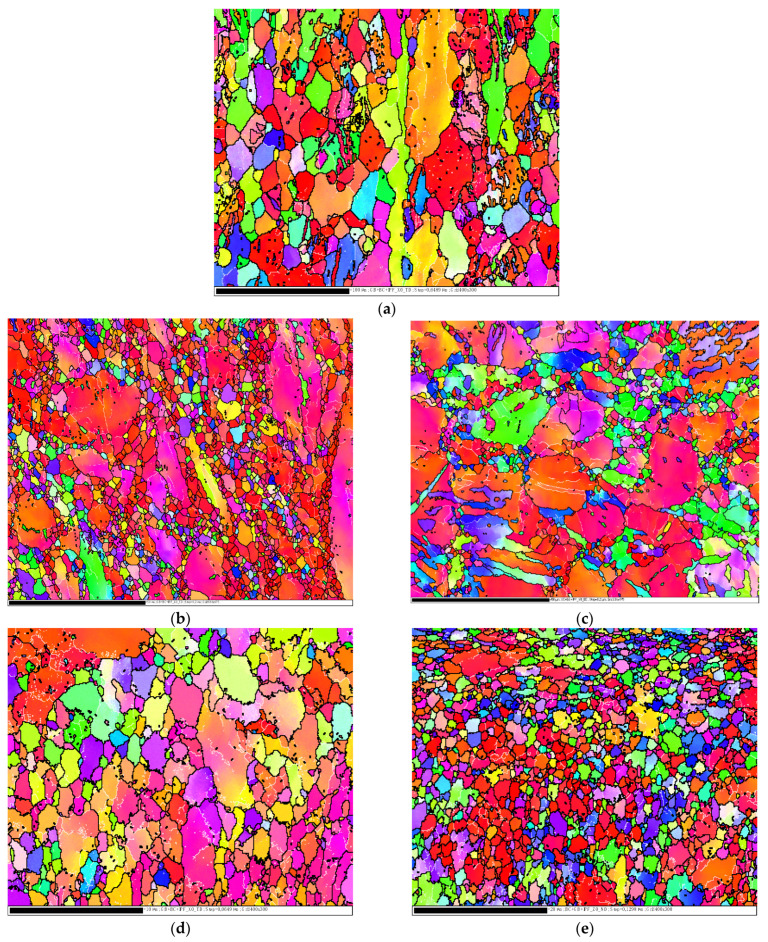
EBSD inverse pole figure (IPF) maps for the AA-Mg (**a**), and after the ECAP processed using route Bc for different passes, (**b**,**c**) 1P, (**d**,**e**) 4P, with different die angle, ϕ= 90° (**b**,**d**) and ϕ = 120° (**c**,**e**).

In the current study, EBSD was used to characterize the microstructural evolution of as-annealed Mg (AA-Mg) after ECAP processing to study how it was affected by the different processing parameters. Figure 2 illustrates the inverse pole figure (IPF) maps of the AA-Mg and the ECAPed samples using route Bc for one pass (1P) and four passes (4P), with different die angles, ϕ = 90° and 120°.

The IPF map of AA-Mg clearly shows that it has inhomogeneous coarse grains, as presented in Figure 2a, after the first ECAP pass grain refinement was observed, regardless of die angle. However, using die angle 90° led to higher refinement, as shown in Figure 2b. This can be argued to relate to the higher strain experienced using the 90° die angle relative to that experienced using the 120° die angle [37]. Table 2 illustrates the grain sizes corresponding to each number of passes and to each die angle through route Bc. Increasing the number of passes up to 4P resulted in even further refinement as the grain size reduced from 1.492 to 0.88 µm using ϕ= 90° and from 2.62 to 1.896 µm using ϕ= 120°.

**Figure 3:**
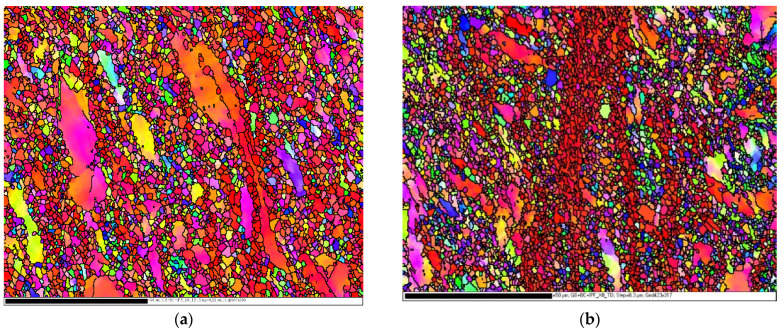
EBSD inverse pole figure (IPF) maps of the AA-Mg after ECAP process for two passes using ϕ= 90° and different routes, route A (**a**) and route C (**b**).

Figure 3 presents the IPF maps of the 2P ECAPed samples using routes A and C. It is shown that the route A sample contains coarser grains than route C as the maximum grain sizes were 22.62 µm and 11.052 µm, respectively. However, the average grain size was approximately the same, 1.492 µm and 1.374 µm, in route A and C, respectively.

**Figure 4:**
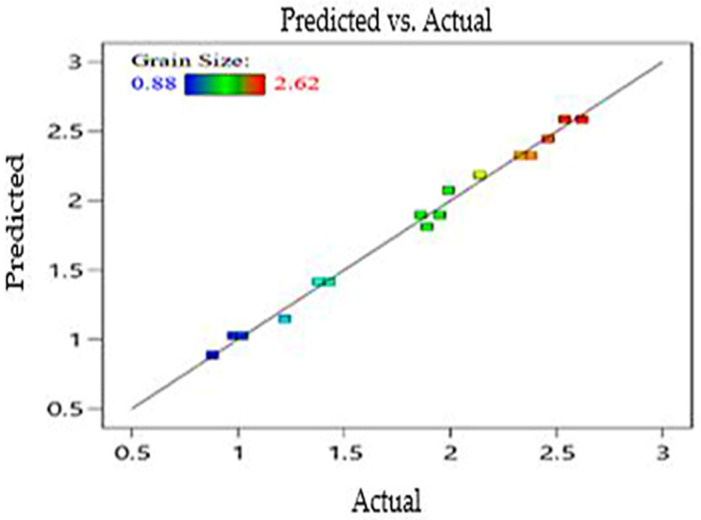
Predicted and actual value of ECAP responses G_R_.

Equation (3) represents the predicted linear model of Mg’s G_R_ based on data from the experiments. The regression coefficient of grain size is (R^2^ > 0.9934), and the adjusted value of R^2^ is 0.9889. Figure 4 presents a comparison between predicted and actual G_R_.

G_R_ = 0.570139 − 0.649646 × N + 0.0204069 × φ − 0.12025 × X_1_ − 0.261167 × X_2_ + 0.00261528 × N × φ + 0.0249236 N^2^ (3)

**Figure 5:**
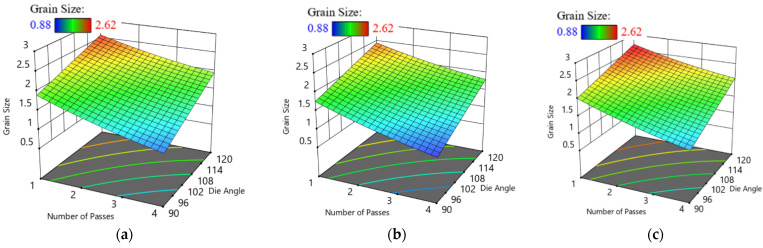
Three-dimensional plot of G_R_ with N and ϕ at route A (**a**), Bc (**b**) and C (**c**).

In addition, the three-dimensional plot, Figure 5, illustrates G_R_ as a function of N and ϕ through the different studied routes. For routes A, Bc and C, the increase in ECAP die angle results in an increase in G_R_, which lies in good agreement with the experimental results. On the contrary, there is an inverse relationship between the number of passes and G_R_. The minimum optimum G_R_ was obtained at four passes and 90° ECAP die angle. The values of G_R_ are 0.98 µm, 0.88 µm and 1.22 µm for route A, Bc and C, respectively, as shown in Figure 5.

### 3.2. Mechanical Properties 

**Figure 6:**
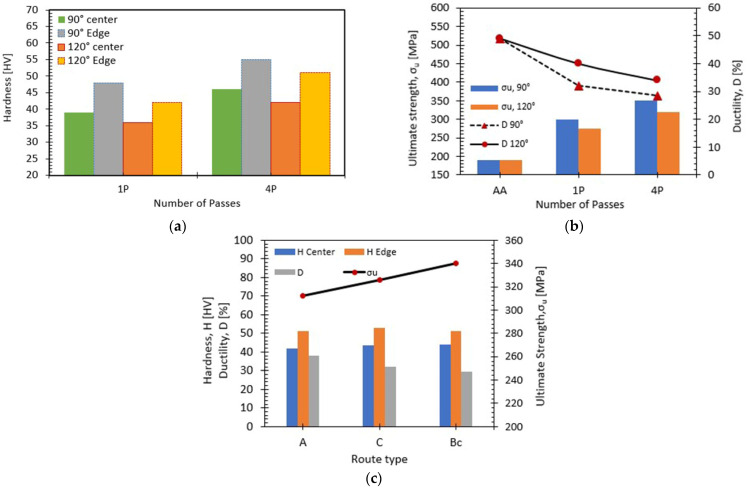
Mechanical properties of the AA-Mg after ECAP process at the different conditions; effect of number of passes and die angle using route Bc (**a**,**b**), and effect different routes, A, C and Bc using ϕ = 90° for two passes (**c**).

Vicker’s microhardness tests across the central and edge regions of the ECAPed billets were used to evaluate the homogeneity of hardness. The hardness values of the ECAPed Mg billets processed via route Bc are plotted in Figure 6a as a function of the number of passes, with the different internal die angles taken into account. Figure 6a shows that the AA-Mg hardness significantly increased with increasing the number of passes in both the central and edge areas. The 90° die recorded higher HV-values in the central and edge regions compared to the ϕ = 120°. ECAP processing through 1P with an internal angle of 90° die revealed an increase in the HV by 84% at the edge and 50% at the central regions compared to the AA-Mg counterpart. This discrepancy in HV increase between the edge and the center could be argued to relate to the friction between the die walls and the sample, which led to more strain hardening. Processing through 1P using 90° die angle showed an increase in the HV by 15% and 8% at the edge and central regions, respectively, compared to ECAP processing with the same conditions using the 120° die, as presented in Figure 6a. The decrease in HV values with increasing ϕ can be argued to relate to the decrease in the plastic strain. Additionally, increasing the plastic strain up to 4Bc using the 90° die angle recorded a significant HV increase at the edge and central areas by 112% and 78%, respectively, compared to the AA-Mg counterpart. Comparatively, the 4Bc condition of the 90° die displayed 8% and 9% increase in the HV-values at the edge and central regions, respectively, compared to the 120° ϕ counterparts. This discrepancy can be argued to relate to the grain refinement mentioned above. Finally, a conclusion can be reached that strain hardening assisted in enhancing the HV-values with increasing the passes number.

Equations (4) and (5) show the predicted modeling of the inverse of H_C_ and H_E_. The regression coefficients of H_C_ and H_E_ are 0.9657 and 0.9741, respectively.

1/H_C_ = 0.0171726 − 0.000110407 × N + 0.000107254 × φ − 0.000901199 × X_1_ − 0.00320891 × X_2_ – 0.00000961846 × N × φ + 0.000507579 × N × X_2_ (4)

1/H_E_ = 0.0159481 − 0.000941798 × N + 0.0000850833 × φ − 0.00033721 × X_1_ − 0.000736064 × X_2_ – 0.000014373 × N × φ + 0.000288905 × N^2^ (5)

**Figure 7:**
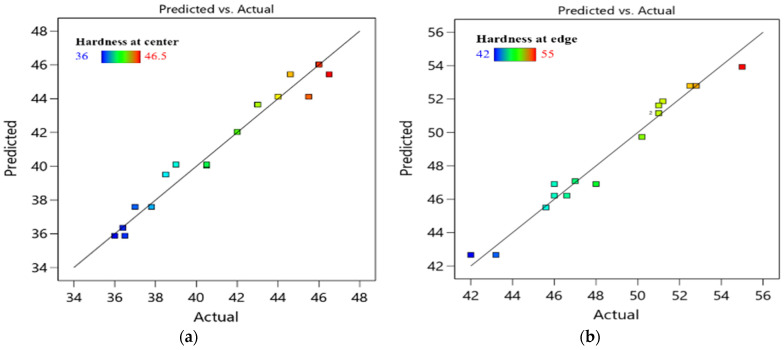
Predicted and actual value of ECAP responses H_C_ (**a**), H_E_ (**b**).

**Figure 8:**
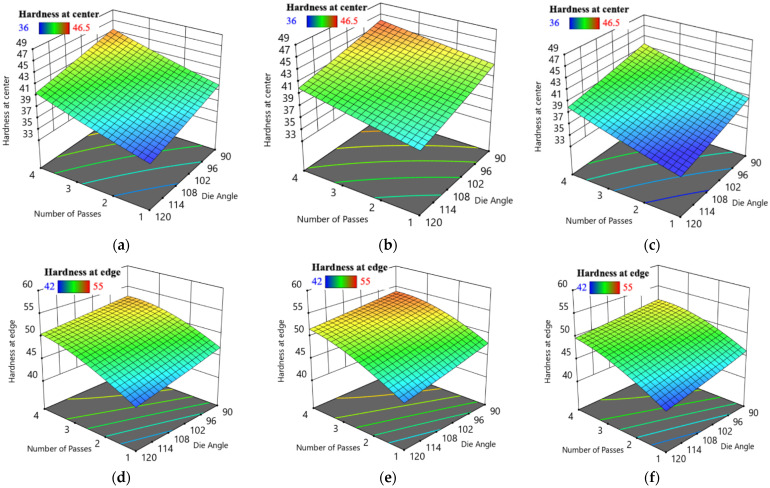
Three-dimensional plot of H_C_ (**a**–**c**) and H_E_ (**d**–**f**) with N and ϕ at route A (**a**,**d**), Bc (**b**,**e**) and C (**d**,**f**).

Figure 7 compares actual and predicted hardness values at central and edge areas. Moreover, Figure 8 shows a three-dimensional response surface plot of H_C_, and H_E_ responses against N and ϕ at a constant processing route. For routes A, Bc and C, the increase in ECAP die angle resulted in a decrease in both H_C_ and H_E_. On the contrary, there is a proportional relationship between the number of passes and both H_C_ and H_E_. The maximum optimum H_C_ and H_E_ were obtained at four passes and 90° ECAP die angle. The optimum H_C_ values are 52.8 HV, 46 HV and 43 HV for route A, Bc and C, respectively, while the maximum values of H_E_ are 46.5 HV, 55 HV and 51.2 HV for route A, Bc and C, respectively. 

Tensile tests were conducted for AA-Mg before and after ECAP processing with the different studied conditions. The ultimate tensile stress (σ_u_) and ductility (D) of the ECAPed samples are plotted in Figure 6b,c. As seen in Figure 2, increasing the ECAP passes results in a significant reduction in grain size, which consequently increases the ultimate strength. Figure 6b shows that, using ϕ = 90°, the 1P sample showed an enhancement in σ_u_ by 58%; however, ductility was decreased by ~ 35% compared to the AA-Mg counterpart. On the other hand, 1P at ϕ = 120° resulted in a rise in σ_u_ by 44%. Increasing the number of ECAP passes up to 4P showed additional enhancement in the σ_u_ by 15% compared to 1P counterpart. The 4Bc condition yielded further improvement in σ_u_ by 16% and a decline in ductility by 10%. Finally, using routes A, C and Bc resulted in increase in σ_u_ by 64%, 71% and 79%, respectively, compared to AA-Mg.

The increase in σ_u_ is a direct consequence of the ultra-fine grains, which can be described using Hughes’ theoretical model [49]. The imposed strain from ECAP processing leads to dislocation motion. As the strain increases, more and more dislocations are absorbed by the low-angle grain boundaries, gradually transforming the low-angle grain boundaries into stable high-angle grain boundaries. Therefore, the grains are refined because of the high-angle grain boundaries formation. However, severe plastic deformation techniques, such as ECAP processing, led to high dislocation density, which delayed the dislocation mobility [50,51,52,53,54,55,56,57], consequently improving the hardness and tensile strength of the ECAPed AA-Mg billets. 

Moreover, the significant grain refinement provides the grain boundary strengthening mechanism, thus enhancing the mechanical properties in good agreement with Ref. [58]. As mentioned above, using ϕ = 90° led to finer grain size compared to 120°, which resulted in higher ultimate strength. However, the ductility reduction obtained in ϕ = 90° and 120° with increasing the number of passes was attributed to smaller grains size, which led to increasing the grain boundary area per unit volume. Consequently, increasing the strengthening results in ductility drop, and similar findings were mentioned in previous work [59]. However, 120° ϕ showed higher ductility compared to 90° die, as presented in Figure 6b, which could be argued that the lower strain occurred when using the 120° die angle compared to ϕ= 90°.

In addition, the tensile responses of Mg specimen are σ_u_ and D%. Equations (6) and (7) represent the linear modelling of tensile response. The regression coefficients of σ_u_ and D% are 0.9948 and 0.9752, respectively.

σ_u_ = −553.75000 + 324.62500 × N + 5.15000 × φ + 736.00000 × X_1_ + 207.50000 × X_2_ − 1.42500 × N × φ – 75.50000 × N × X_1_ − 49.75000 × N × X _2_– 4.82500 × φ × X_1_ – 22.87500 × N^2^ (6)

D = 68.26667 − 8.10000 × N − 0.353333× φ − 6.90000× X_1_ − 13.40000 × X_2_ + 0.143333 × N × φ + 0.056667 × φ × X_1_ + 0.103333 × φ × X_2_ − 1.41667 × N^2^ (7)

**Figure 9:**
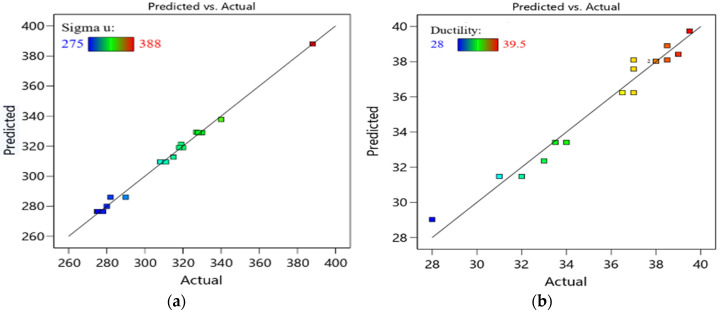
Predicted and actual value of ECAP responses σ_u_ (**a**) and D (**b**).

**Figure 10:**
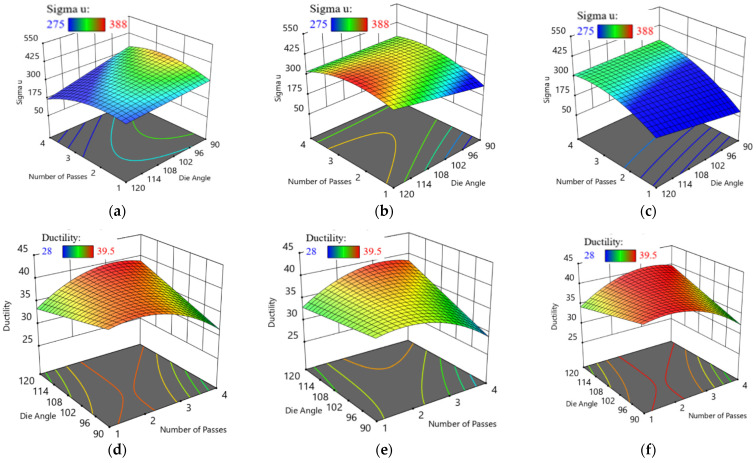
Three-dimensional plot of σ_u_ (**a**–**c**) and D (**d**–**f**) with N and ϕ at route A (**a**,**d**), Bc (**b**,**e**) and C (**c**,**f**).

The relationships between the actual and predicted responses σ_u_ and D are shown in Figure 9. In addition, Figure 10 shows the three-dimensional interaction effect of ECAP parameters on σ_u_ and D while generating interaction graphs and response surface plots between two variables at constant processing route. For route A, Bc and C, there are slight effects of ECAP die angle on σ_u_ of specimen. However, there are different relations between the number of ECAP passes and σ_u_ in each route. For route A, increasing ECAP number of passes will decrease the σ_u_. The maximum σ_u_ at route A (330 MPa) was obtained at four passes and 90° die angle. 

Moreover, there is a proportional relationship between N and σ_u_ at route Bc until we reach two passes, and a decrease in σ_u_ occurs. The maximum σ_u_ at route Bc (388 MPa) was obtained at two passes and 120° die angle. Finally, for route C, the maximum σ_u_ at route C (327 MPa) was obtained at four passes and 90° die angle. For route A, Bc and C, there are slight effects of ECAP die angle on D of specimen. However, there are different relations between the number of ECAP passes and D in each route. For route A, Bc and C, there is a proportional relationship between N and D until we reach two passes, and a decrease in D occurs. The minimum D values at route A, Bc and C are 31%, 28% and 39.5%, respectively. The optimum D for all three processing routes was obtained at four passes and 90° die angle. 

Furthermore, analysis of variance (ANOVA) is used to examine independent parameters, N, φ, X_1_ and X_2_, in order to determine which ones have a significant impact on performance parameters, G_R_, H_C_, H_E_, σ_u_ and D [60]. The significant effects with *p*-values less than 0.05 indicate that the independent parameters, as well as the individual model coefficients and interaction terms, are statistically different from zero at the 95% confidence level [61]. Table 3 presents the significant parameters of the five responses after eliminating the insignificant effects using multiple regression analysis on experimental data.

Table 4 presents the statistical tests, namely F-value, *p*-value, lack of fit, adequate precision, regression coefficient (R^2^), adjusted R^2^ and predicted R^2^ of the five models. The F-value is the difference between the variation attributed to individual factors and the variance due to the error term. F-values of ECAP responses greater than 4 indicate that changing an input ECAP parameter has a significant impact on the response quality criterion [62]. Die angle has the greatest impact on G_R_, H_C_ and D. Otherwise, number of passes and route type have a significant effect on H_E_ and σ_u_, respectively. 

The model significant (*p*-values) are less than 0.05 for all ECAP responses, indicating that the independent parameters, as well as the individual model coefficients and interaction terms, are statistically different from zero at the 95% confidence level. The ECAP responses’ lack of fit is greater than 0.05, implying that the model is good [63]. The signal to noise (S/N) ratio is computed with “adequate precision” to determine the model’s validity. It is recommended that the ratio exceed four [62]. The ECAP responses’ adequate precision is greater than four and indicates that there is sufficient signal and the model can be applicable to navigate the design space. The regression coefficient R^2^ is high and indicates that the ECAP responses’ model created by the experiment is desirable. The adjusted R^2^ for the five responses is close to the predicted coefficient R^2^. 

### 3.3. Optimization Results

#### 3.3.1. RSM Results

**Figure 11:**
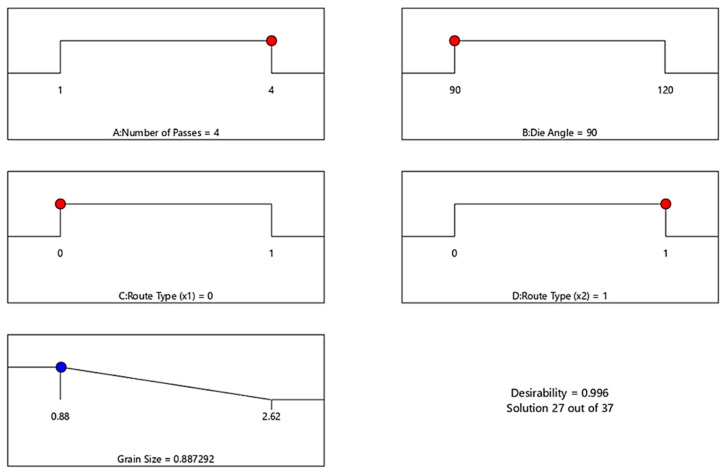
Optimal ECAP process parameter of G_R_. Red dots: ECAP condition. Blue dots: ECAP response.

This section presented the optimal ECAP condition of the desire response. Stat-Ease Design Expert software (version 13.0.5) is a useful tool for optimizing the ECAP condition. Figure 11 shows the optimal G_R_ response of ECAP process and corresponding conditions. For all the following optimization findings the red dot and the blue dotes the indicated the ECAP processing condition and ECAP response, respectively. The optimization target is set to “In range”, and the solution destination is set to “Minimize.” The desirability function’s predicted output is in the form of “smaller-is-better” characteristics. The optimal process condition values include number of passes (A) = 4 passes, die angle (B) = 90°, dummy variable X_1_(C) = 0 and dummy variable X_2_(D) = 1 for minimum G_R_ value of 0.8872 µm. The optimum value of G_R_ was obtained in the range between 0.88 and 2.62 µm. 

**Figure 12:**
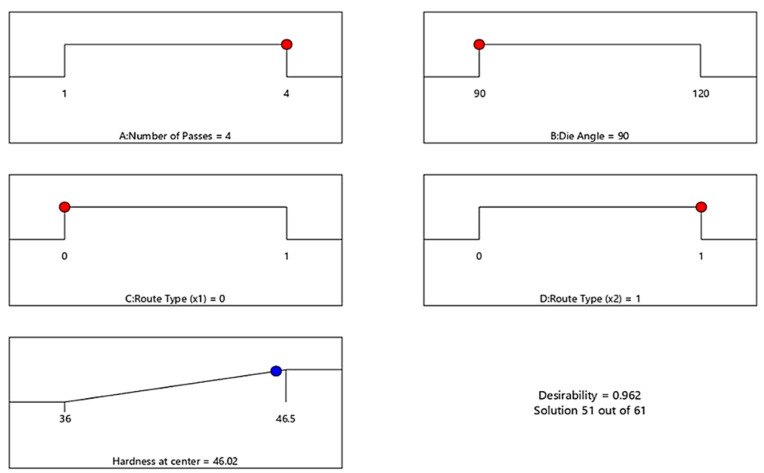
Optimal ECAP process parameter of H_c_. Red dots: ECAP condition. Blue dots: ECAP response.

In addition, the optimal H_C_ response of ECAP process and corresponding conditions is presented in Figure 12. The optimization target is set to “In range”, and the solution destination is set to “Maximize.” The desirability function’s predicted output is in the form of “larger-is-better” characteristics. The optimal process condition values include number of passes (A) = 4 passes, die angle (B) = 90°, dummy variable X_1_(C) = 0 and dummy variable X_2_(D) = 1 for maximum H_C_ value of 46.02 HV. The optimum value of H_C_ was obtained in the range between 36 and 46.5 HV. 

**Figure 13:**
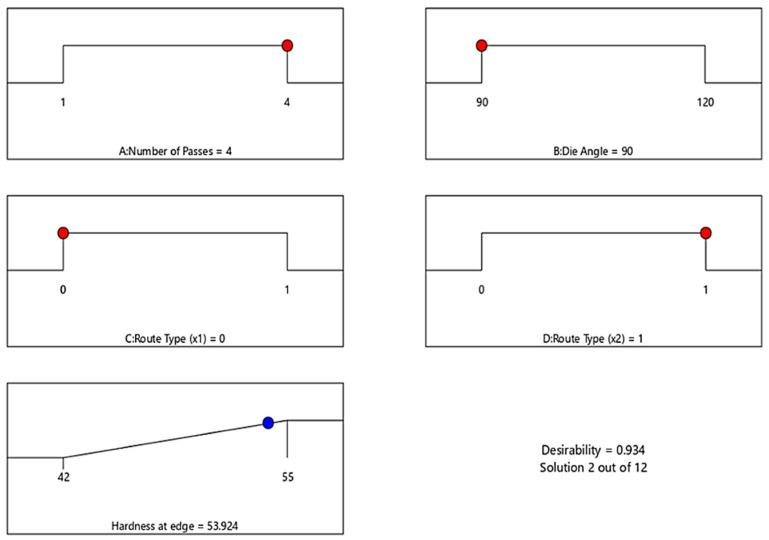
Optimal ECAP process parameter of H_E_. Red dots: ECAP condition. Blue dots: ECAP responce.

Furthermore, Figure 13 illustrates the optimal H_E_ response of ECAP process and corresponding conditions. The optimization target is set to “In range”, and the solution destination is set to “Maximize.” The desirability function’s predicted output is in the form of “larger-is-better” characteristics. The optimal process condition values include number of passes (A) = 4 passes, die angle (B) = 90°, dummy variable X_1_(C) = 0 and dummy variable X_2_(D) = 1 for maximum H_E_ value of 53.924 HV. The optimum value of H_E_ was obtained in the range between 42 and 55 HV. 

**Figure 14:**
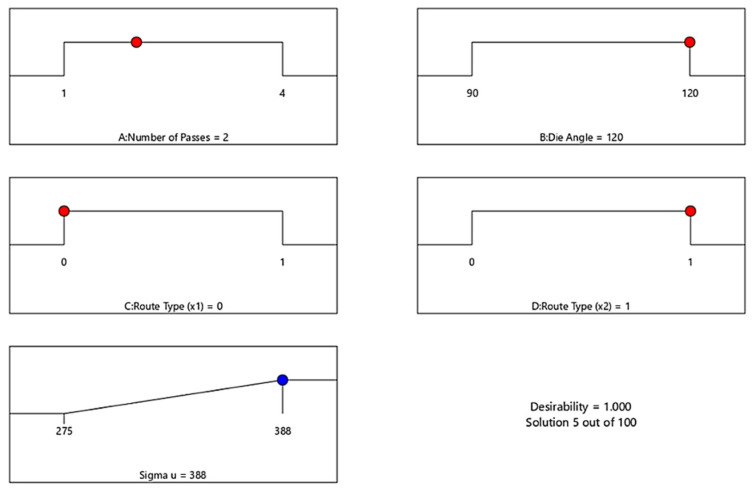
Optimal ECAP process parameter of σ_u_. Red dots: ECAP condition. Blue dots: ECAP response.

Figure 14 presents the optimal σ_u_ response of ECAP process and corresponding conditions. The optimization target is set to “In range”, and the solution destination is set to “Maximize.” The desirability function’s predicted output is in the form of “larger-is-better” characteristics. The optimal process condition values include number of passes (A) = 2 passes, die angle (B) = 120°, dummy variable X_1_(C) = 0 and dummy variable X_2_(D) = 1 for maximum σ_u_ value of 388 MPa. The optimum value of σ_u_ was obtained in the range between 275 and 388 MPa.

**Figure 15:**
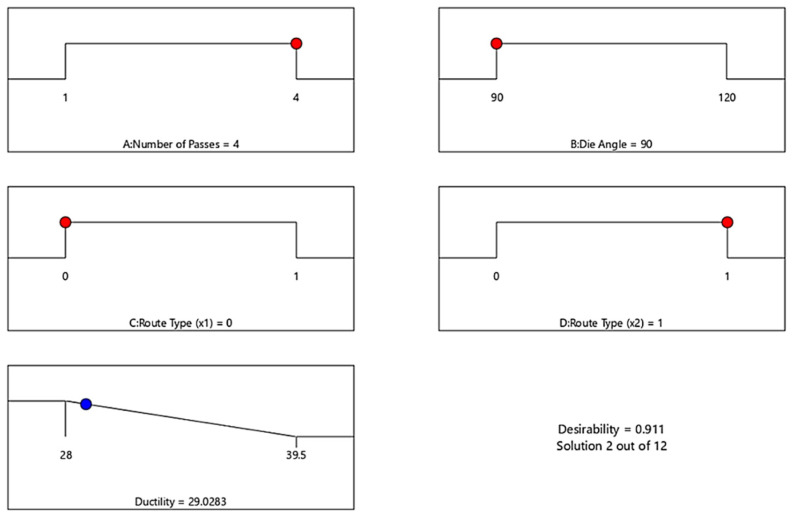
Optimal ECAP process parameter of D. Red dots: ECAP condition. Blue dots: ECAP response.

Finally, Figure 15 shows the optimal D response of ECAP process and corresponding conditions. The optimization target is set to “In range”, and the solution destination is set to “Minimize.” The desirability function’s predicted output is in the form of “smaller-is-better” characteristics. The optimal process condition values include number of passes (A) = 4 passes, die angle (B) = 90°, dummy variable X_1_(C) = 0 and dummy variable X_2_(D) = 1 for minimum D value of 29.0283%. The optimum value of D was obtained in the range between 28 and 39.5%. For a confirmation test, these optimum conditions of ECAP and responses are compared to the GA results obtained in the next step.

#### 3.3.2. GA and Hybrid RSM-GA Results

Genetic algorithm (GA) used to determine the optimum set of the ECAP independent variables that contribute to the lowest possible G_R_ and D and the higher possible H_C_, H_E_ and σ_u_. Each ECAP response proposed in Equation (3) to Equation (7) is taken as the objective function and subjected to the ECAP boundary condition, N, φ, X_1_ and X_2_ by using genetic algorithm can be expressed as: 

Minimize ECAP (number of passes. ECAP die angle, X_1_, X_2_). Subjected to ranges of ECAP condition;

1 ≤ N ≤ 4 (pass)

90 ≤ φ ≤ 120 (°)

X_1_ϵ [0,1]

X_2_ ϵ [0,1]

**Figure 16:**
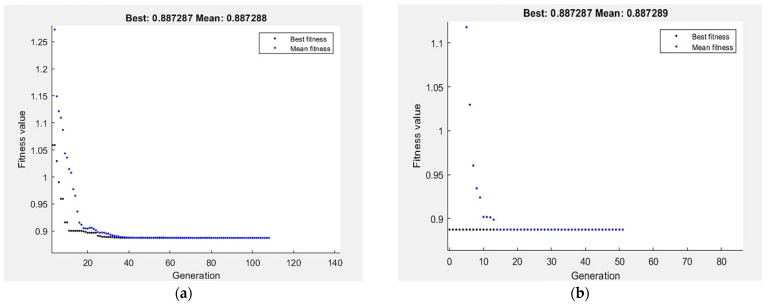
Optimum G_R_ by GA (**a**), and hybrid RSM-GA (**b**).

For GA optimization technique, the performance of fitness value and run solver view results from MATLAB show the best G_R_ and corresponding ECAP conditions. The best value of G_R_ by GA is 0.8872 µm obtained at four passes, 90° die angle and route Bc for N, φ and processing route type, respectively, as shown in Figure 16. In order to improve the results of GA, hybrid of response surface methodology and GA (RSM-GA) was performed. An initial population of hybrid RSM-GA based on RSM optimum ECAP condition of N, φ, X_1_ and X_2_ are four passes, 90°, 0 and 1, respectively. The minimum optimum G_R_ by hybrid RSM-GA is 0.8872 µm obtained at four passes, 90° and route Bc. 

**Figure 17:**
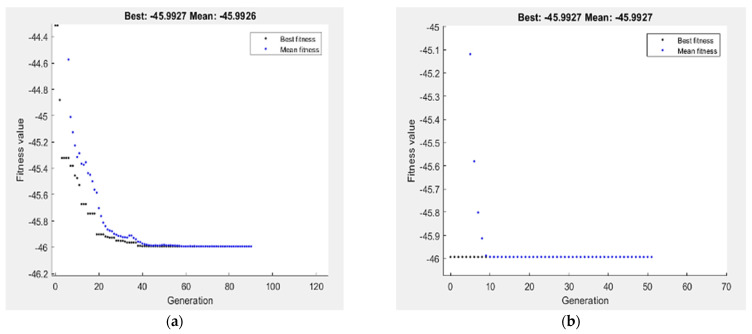

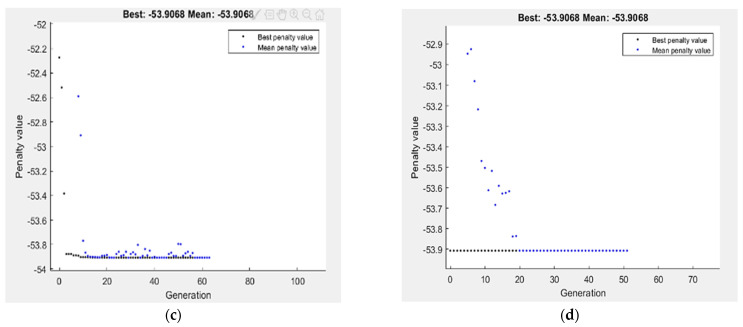
Optimum H_C_ (**a**,**b**), and H_E_ (**c**,**d**) by GA (**a**,**c**), and hybrid RSM-GA (**b**,**d**).

The optimization of hardness response by GA is presented in Figure 17. The maximization of H_C_ and H_E_ proposed in Equations (4) and (5) is taken as the fitness function and subjected to the ECAP boundary condition. The best value of H_C_ and H_E_ by GA is 45.9927 HV and 53.9068 HV, respectively, obtained at four passes, 90° die angle and route Bc. Hybrid (RSM-GA) of H_C_ and H_E_ was performed to improve the results of GA. The maximum optimum H_C_ and H_E_ by hybrid RSM-GA are 45.9927 HV and 53.9068 HV, respectively, obtained at four passes, 90° die angle and route Bc, as shown in Figure 17.

**Figure 18:**
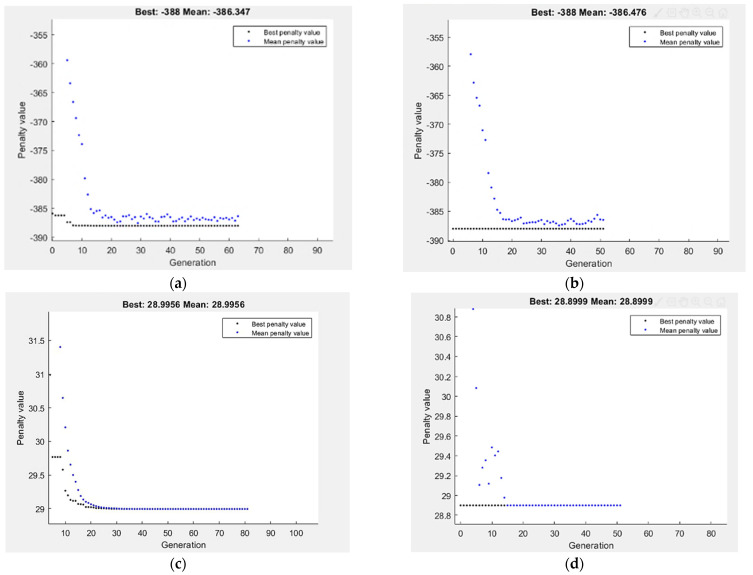
Optimum σ_u_ (**a**,**b**), and D (**c**,**d**) by GA (**a**,**c**), and hybrid RSM-GA (**b**,**d**).

The optimization of tensile response, σ_u_ and D, by GA is presented in Figure 18. The maximization of σ_u_ proposed in Equation (6) is taken as the fitness function and subjected to the ECAP boundary condition. The best value of σ_u_ by GA is 388 MPa obtained at two passes, 120° die angle and route Bc. 

On the other hand, the minimization of D% proposed in Equation (7) is taken as the fitness function and subjected to the ECAP boundary condition. The best value of D% by GA is 28.99% obtained at four passes, 90.29° die angle and route Bc. The maximum σ_u_ by hybrid RSM-GA is 388 MPa obtained at two passes, 120° die angle and route Bc. Moreover, minimum optimum D% by hybrid RSM-GA is 28.899 % obtained at four passes, 90° die angle and route Bc, as shown in Figure 18. Table 5 summarized the comparison of ECAP responses values at experimental, RSM, GA and hybrid RSM-GA. 

#### 3.3.3. Validation of GA

This section proposed the optimal ECAP parameters of different responses, namely G_R_, H_C_, H_E_, σ_u_ and D. The presented optimal ECAP parameters, such as N, φ and processing route type, are based on previous studies of pure Mg that recommended the number of passes from one to twelve passes and ECAP die angle from 70° to 135° [64,65,66,67]. Table 6 presents the optimal condition of ECAP process of different responses by genetic algorithm (GA) and hybrid RSM and GA.

## 4. Conclusions

Billets of pure Mg were processed through ECAP up to four passes of routes Bc, A and C using two dies with channel angles of 90° and 120° at a temperature of 225 °C. 

Many empirical models were developed to assess the effect of ECAP processing parameters on the microstructural evolution and mechanical properties of Mg billets. The following conclusions can be extracted:

1. ECAP parameters of four passes, ϕ = 90° and route Bc produce the most significant grain refinement.
2. 4-Bc experienced a significant reduction in the grain size by 86% compared to the as-annealed counterparts.
3. ECAP parameters of four passes, ϕ = 90° and route Bc resulted in the best Vicker’s microhardness values at both the central and the peripheral regions.
4. 4-Bc processing through the 90° die angle recorded a significant HV increase at the edge and central areas by 112% and 78%, respectively, compared to the as-annealed counterpart.
5. ECAP parameters of two passes, ϕ = 120° and route Bc resulted in the highest ultimate tensile strength.
6. ECAP parameters of four passes, ϕ = 90° showed the most enhancement in the ductility at fracture of the Mg billets.

## Figures and Tables

**Table 1:** ECAP parameters levels.

ECAP Parameters	Parameters Levels		
	L1	L2	L3
Number of passes (N)	1	2	4
ECAP die angle (ϕ)	90	120	
Processing route type	A	Bc	C

**Table 2:** Grain size (G_R_) of AA-Mg and after ECAP process through route Bc using two different die angles (ϕ).

Data, µm	AA-Mg	Route Bc			
		1P		4P	
		ϕ 90°	ϕ 120°	ϕ 90°	ϕ 120°
Min. grain size	1.106	0.507	0.505	0.21	0.81
Max. grain size	34.02	9.291	22.727	7.14	20.15
Average grain size	6.338	1.492	2.62	0.88	1.89
Standard deviation	5.815	0.945	1.433	0.73	1.11

**Table 3:** Significant independent parameters for each ECAP response.

		Significant Effect
Response	G_R_	N, φ, X_1_, X_2_, N φ
	H_C_	N, φ, X_2_
	H_E_	N, φ, X_2_, N φ, N^2^
	σ_u_	φ, X_1_, X_2_, N φ, N X_1_, N X_2_, φ X_1_, N^2^
	D	φ, X_2_, N φ, φ X_2_, N^2^

**Table 4:** Statistical tests of ECAP responses.

Response	F-Value (F > 4)	Model Significant (*p* < 0.05)	Lack of Fit (*p* > 0.05)	Adeq Precision (Ratio > 4)	R^2^	Adjusted R^2^	Predicted R^2^
G_R_	224.52	<0.0001	0.1395	41.3955	0.9934	0.9889	0.968
H_C_	42.22	<0.0001	0.4739	17.4906	0.9657	0.9428	0.895
H_E_	56.44	<0.0001	0.6959	22.39	0.9741	0.9569	0.9063
σ_u_	126.59	<0.0001	0.1939	42.7681	0.9948	0.9869	0.9894
D	34.41	<0.0001	0.1372	18.5288	0.9752	0.9469	0.9553

**Table 5:** Summary results of pure Mg ECAP process.

Response		Experimental	RSM	GA	RSM-GA
G_R_	Value	0.88	0.8872	0.8872	0.887287
	Cond.	4passes, 90°, Route Bc	4passes, 90°, Route Bc	4passes, 90°, Route Bc	4passes, 90°, Route Bc
H_C_	Value	46.5	46.02	45.9927	45.9927
	Cond.	4passes, 90°, Route A	4passes, 90°, Route Bc	4passes, 90°, Route Bc	4passes, 90°, Route Bc
H_E_	Value	55	53.924	53.9068	53.9068
	Cond.	4passes, 90°, Route Bc	4passes, 90°, Route Bc	4passes, 90°, Route Bc	4passes, 90°, Route Bc
σ_u_	Value	388	388	388	388
	Cond.	2passes, 120°, Route Bc	2passes, 120°, Route Bc	2passes, 120°, Route Bc	2passes, 120°, Route Bc
D	Value	28	29.0283	28.9956	28.899
	Cond.	4passes, 90°, Route Bc	4passes, 90°, Route Bc	4passes, 90°, Route Bc	4passes, 90°, Route Bc

**Table 6:** Validated ECAP response based on previous studies.

Response		GA	RSM-GA
G_R_ (µm)	Value	0.269927	0.269927
	Cond.	4passes, 70°, Route Bc	4passes, 70°, Route Bc
H_C_ (HV)	Value	47.4951	47.4951
	Cond.	4passes, 80°, Route Bc	4passes, 80°, Route Bc
H_E_ (HV)	Value	54.7207	54.7207
	Cond.	4passes, 80°, Route Bc	4passes, 80°, Route Bc
σ_u_ (MPa)	Value	422.5	422.5
	Cond.	2passes, 135°, Route Bc	2passes, 135°, Route Bc
D (%)	Value	4.0846	4.0846
	Cond.	6passes, 80°, Route Bc	6passes, 80°, Route Bc

## Data Availability

All the raw data supporting the conclusion of this paper were provided by the authors.

## Nomenclature

ECAP	equal channel angular pressing
RSM	response surface methodology
ANOVA	analysis of variance
HCP	hexagonal close-packed
SPD	severe plastic deformation
UFG	ultra-fine grain
$\varepsilon_{eq}$	the equivalent strain
ϕ	ecap die angle
Ψ	outer corner angle
N	number of passes
FESEM	field emission scanning electron microscope
EBSD	Electron back-scatter diffraction
1P and 4P	one pass and 4 passes
AA	as-annealed
IPF	inverse pole figure
Hv	vicker’s microhardness
CCD	central composite design
DF	desirability function
GA	genetic algorithm
FDM	fused deposition modeling
GA-ANN	genetic algorithm-artificial neural network
GA-RSM	genetic algorithm-response surface methodology
GA-ANFIS	genetic algorithm-adaptive neuro fuzzy interface system
G_R_	grain size
H_C_	hardness measurement at center
H_E_	hardness measurement at edge
σ_u_	ultimate tensile strength
D	ductility
X_1_ and X_2_	dummy variables
R^2^	regression coefficient
S/N	signal to noise

## Appendix A

**Table A1:** Design of experiment of ECAP parameters and their response.

Run	ECAP Parameters			Response				
	A	B	C	Grain Size G_R_ (μm)	Hardness		Tensile Strength	
	N	φ	Route Type		H_E_ (HV)	H_C_ (HV)	σ_u_ (MPa)	D (%)
1	1	120	A	2.62	42	36	278	33.5
2	2	120	A	2.33	46.6	37	282	38
3	4	90	C	1.22	51.2	43	327	33
4	2	120	C	2.46	45.6	36.4	280	38.5
5	2	90	Bc	1.38	51	45.5	320	36.5
6	2	120	A	2.38	46	37.8	290	38
7	2	90	Bc	1.43	51	44	318	37
8	4	120	Bc	1.89	51	42	319	39
9	4	120	C	1.99	50.2	40.5	315	39.5
10	2	120	Bc	2.14	47	38.5	388	37
11	1	120	A	2.54	43.2	36.5	275	34
12	4	90	Bc	0.88	55	46	340	28
13	1	90	A	1.95	48	39	308	38.5
14	4	90	A	0.98	52.5	46.5	330	31
15	4	90	A	1.02	52.8	44.6	328	32
16	1	90	A	1.86	46	40.5	311	37
